# Sensitive and early detection of mitochondrial dysfunction in the liver of NASH model mice by PET imaging with ^18^F-BCPP-BF

**DOI:** 10.1186/s13550-018-0420-6

**Published:** 2018-07-16

**Authors:** Toshihiro Sakai, Hiroyuki Ohba, Shingo Nishiyama, Takeharu Kakiuchi, Osamu Inoue, Hideo Tsukada

**Affiliations:** 1 Hot Laboratory, Hanwa Intelligent Medical Center, Hanwa Daini Senboku Hospital, 3176 Fukai Kitamachi, Naka-ku, Sakai, Osaka 599-8271 Japan; 2 Central Research Laboratory, Hamamatsu Photonics KK, 5000 Hirakuchi, Hamakita-ku, Hamamatsu, Sizuoka 434-8601 Japan

**Keywords:** NASH mouse, Mitochondrial dysfunction, Mitochondrial complex-I, PET, ^18^F-BCPP-BF

## Abstract

**Background:**

Nonalcoholic fatty liver disease is a common disorder that progresses from simple fatty liver (steatosis) to nonalcoholic steatohepatitis (NASH). It is thought that mitochondrial dysfunction plays a critical role in the progression of NASH. In this study, we developed a non-invasive method for early diagnosis and staging of NASH that directly measures mitochondrial complex-I (MC-I) activity in the liver of NASH model mice by positron emission tomography (PET) imaging using the novel tracer 2-tert-butyl-4-chloro-5-[6-(4-[^18^F]fluorobutoxy)-pyridin-3-ylmethoxy]-2H-pyridazin-3-one (^18^F-BCPP-BF). Liver uptake of ^18^F-BCPP-BF in NASH and age-matched control mice was measured as a standard uptake value over a period of 1 to 12 weeks. Histopathological evaluation of the liver tissue was performed by haematoxylin and eosin staining, and fibrosis was assessed by Masson’s trichrome staining.

**Results:**

Significant mitochondrial dysfunction was detected as early as 1 week after commencing the diet, and MC-I activity in the liver measured by PET was reduced by > 50% relative to that in age-matched control mice after 6 weeks. Liver uptake of ^18^F-BCPP-BF was low throughout the 12-week experimental period. Histopathological examination revealed that steatosis, inflammation, and ballooning progressed from 1 to 6 weeks, with fibrosis observed from 6 to 12 weeks.

**Conclusions:**

PET scans and histopathological analysis revealed that mitochondrial dysfunction in the liver contributed to the progression of NASH. PET imaging with ^18^F-BCPP-BF is a useful tool for detecting NASH at early stages and for monitoring therapeutic response.

## Background

Nonalcoholic fatty liver disease (NAFLD) is a common liver disorder that constitutes a major public health burden worldwide [1]. NAFLD can progress from simple fatty liver (steatosis) to nonalcoholic steatohepatitis (NASH), hepatic cirrhosis, and hepatocellular carcinoma [2]. NASH is characterised histologically by macrosteatosis, inflammation, hepatocyte ballooning, and premicellar fibrosis [3]. Although its pathogenesis is not well understood, there is accumulating evidence suggesting that mitochondrial dysfunction plays a critical role in NAFLD development and progression [4–7]. Mitochondrial dysfunction impairs fatty acid homeostasis and induces the overproduction of reactive oxygen species (ROS) that cause lipid peroxidation, cytokine release, and cell death [8]. However, little is known about how hepatic mitochondrial dysfunction leads to NASH progression [9]. Invasive liver biopsy is currently the gold standard for diagnosing NASH [10]. Therefore, a non-invasive imaging method or biomarkers that would allow NAFLD staging are needed in both clinical practice and basic research to elucidate the pathological mechanisms of NASH and evaluate the efficacy of new therapeutics [11, 12].

We have previously developed novel positron emission tomography (PET) probes with different lipophilicity and affinity for mitochondrial complex-I (MC-I) [13–18]. The BCPP compounds BCPP-BF, BCPP-EF, and BCPP-EM (2-tert-butyl-4-chloro-5-[6-(4-fluorobutoxy)-pyridin-3-ylmethoxy]-2H-pyridazin-3-one (BCPP-BF) and its analogues, BCPP-EF and BCPP-EM) were found to be MC-I selective inhibitors in binding experiments that employed ^3^H-dihydrorotenone in vitro [19]. We also recently succeeded in imaging hepatic mitochondrial dysfunction induced by acetaminophen using 2-tert-butyl-4-chloro-5-[6-(4-[^18^F]fluorobutoxy)-pyridin-3-ylmethoxy]-2H-pyridazin-3-one (^18^F-BCPP-BF) as tracer. PET imaging with ^18^F-BCPP-BF is highly sensitive to changes in mitochondrial function in the liver of live animals [20]. A significant decline in liver uptake of ^18^F-BCPP-BF was observed in rats just 2 h after treatment with a relatively small amount of acetaminophen (100 mg/kg i.p.) without any alterations in plasma alanine aminotransferase and aspartate aminotransferase. Pre-administration of rotenone (0.1 mg/kg/h), a specific MC-I inhibitor, markedly decreased liver uptake of ^18^F-BCPP-BF, indicating that liver uptake of this probe was mainly due to specific binding to MC-I. These results suggest that this method can be used to measure mitochondrial dysfunction during NAFLD progression with high sensitivity.

The aim of this study was to evaluate the effectiveness of PET imaging in detecting MC-I activity for the diagnosis and staging of NAFLD. The methionine-choline-deficient diet (MCD) is one of the most common tools inducing NAFLD in many studies [21]. There are however two drawbacks associated with the use of MCD in mice: dramatic weight loss and lack of insulin resistance [22], which are well-known symptoms in patients with NASH. Severe loss of body weight in mice is associated with increased risk of death; therefore, the use of MCD model is problematic for long-term experiments. Here, we used an improved mouse model of NASH, which was induced by the choline-deficient, l-amino acid defined, high-fat diet (CDAHFD). This diet leads to rapid development of fibrosis without severe loss of body weight [23]. We performed PET imaging of ^18^F-BCPP-BF uptake in the liver and examined associated pathological changes in mice fed CDAHFD for 1–12 weeks.

## Methods

### Animals and reagents

The following experiments were approved by the Ethical Committee of the Central Research Laboratory of Hamamatsu Photonics (approval number HPK2017-04). Control male C57BL/6J and NASH model mice (6–17 weeks old; body weight, 12–25 g) were purchased from Charles River Japan (Yokohama, Japan) and housed for 1 week at 24 ± 1 °C at 12 h:12-h light/dark cycle. The mice were fed A06071302 (60 kcal % fat) (EPS Ekishin Co., Tokyo, Japan) as high-fat diet (HFD) or Certified Rodent Diet 5002 (Japan SLC, Hamamatsu, Japan) as normal diet prior to the experiments. Isoflurane was purchased from Dainippon Pharmaceutical (Osaka, Japan). The precursor of ^18^F-BCPP-BF and the standard compound were obtained from the NARD Institute (Amagasaki, Japan). ^18^F-BCPP-BF was radiolabelled by nucleophilic ^18^F fluorination of its precursor as previously described [13]. Radiochemical purity of ^18^F-BCPP-BF was over 99%, and specific radioactivity was 41.2 ± 7.7 GBq/μmol.

### PET measurements

Four to six animals per group were used for PET imaging. After measuring body weight, the mice were anaesthetised with 1.5–2.0% isoflurane in O_2_, positioned prone on a fixation plate, and placed in the gantry hole of the PET scanner (SHR-38000; Hamamatsu Photonics, Hamamatsu, Japan). After a transmission scan for 15 min using a 68Ge-68Ga rotation rod source, ^18^F-BCPP-BF at 3.3 ± 2.1 MBq (mean ± SD) was intravenously injected into the mouse via tail vein, followed by an emission scan with list mode data acquisition for 90 min. Body temperature was monitored throughout the experiment using a Thermo-Hygro Recorder (TR-71wf; T&D Corporation, Matsumoto, Japan).

PET data were reconstructed using the dynamic row-action maximum likelihood algorithm with a Gaussian filter of 1.0 mm full width at half-maximum and attenuation correction using transmission scan data [24]. Images were acquired every minute for the time-activity curve (TAC), and summation images from the early and late (20–40 and 40–60 min after ^18^F-BCPP-BF injection, respectively) phases were used for reconstruction.

^18^F-BCPP-BF uptake in the liver was high and relatively stable in the early phase in the control condition, and the inhibitory effects of rotenone on ^18^F-BCPP-BF uptake were comparable between early and late phases [20]. We therefore applied averaged data to the early phase to assess the degree of mitochondrial dysfunction in the liver of NASH model mice. Standard uptake value (SUV) was calculated as follows:

$$ \mathrm{SUV}={C}_T\ {W}_s/{D}_{\mathrm{inj}} $$

where *C*_*T*_ is tissue radioactivity (Bq/ml) obtained from PET images, *W*_*s*_ is body weight (g) of the mouse, and *D*_inj_ is the injected dose (Bq) of ^18^F-BCPP-BF. Volumes of interest (VOIs) were defined from summation images of the liver in the early and late phases, and TACs were obtained from dynamic image data as SUV by setting late-phase VOIs.

### Histological examination

At the end of PET scans, mice were sacrificed by exsanguination under isoflurane anaesthesia. The left and right liver lobes were fixed in 10% formalin and cut into 5-μm sections that were stained with haematoxylin and eosin (H&E). Hepatic fibrosis was evaluated by Masson’s trichrome staining (Sigma-Aldrich, St. Louis, MO, USA) according to the manufacturer’s instructions. Liver tissue samples were assigned an NAFLD activity score [25].

### Statistical analysis

Results are expressed as the mean ± standard deviation (SD). Statistical significance was assessed using the Student’s unpaired *t* test. All statistical analyses were conducted with a significance level of *α* = 0.05 (*P* < 0.05).

## Results

### PET measurement

**Fig. 1:**
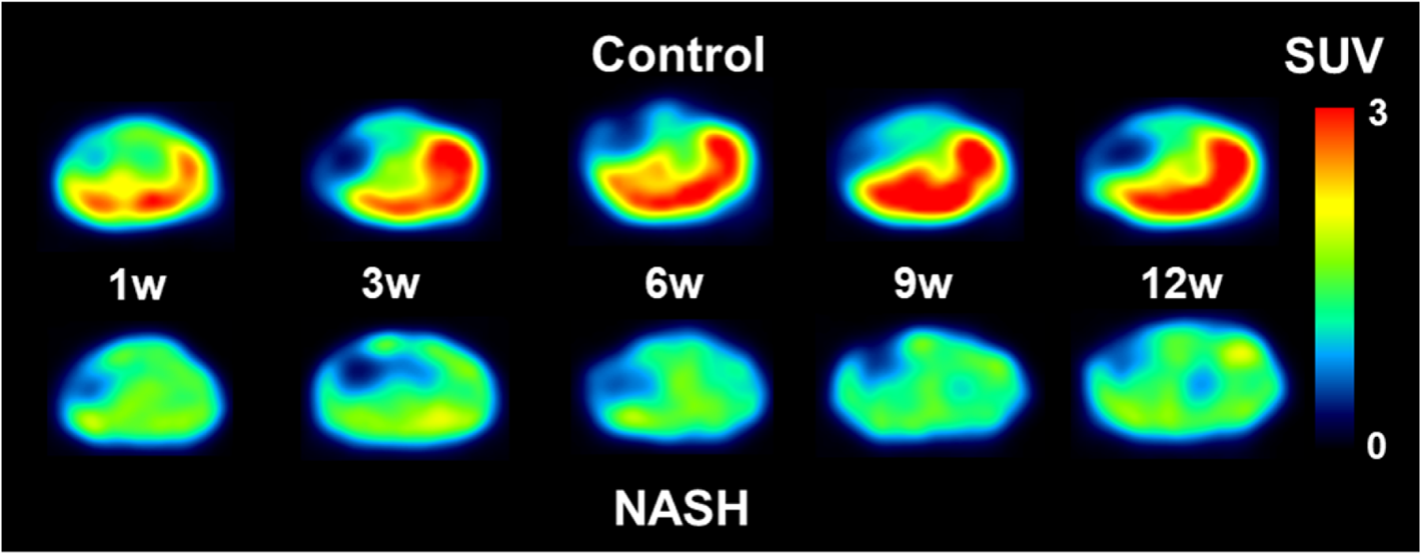
PET images of the liver of mice fed choline-deficient, l-amino acid defined, high-fat diet and of age-matched control mice. PET scanning was conducted for 90 min following injection of mice with ^18^F-BCPP-BF. PET data from 20 to 40 min were reconstructed to obtain images that were used to calculate standard uptake value (SUV)

**Fig. 2:**
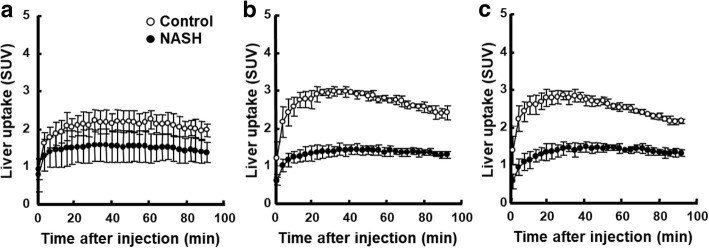
Time course of radioactivity concentration (time-activity curve, TAC) in the liver of mice fed choline-deficient, l-amino acid defined, high-fat diet for 1 (**a**), 6 (**b**), and 9 (**c**) weeks and of age-matched control mice. Data are presented as the mean ± standard deviation of 4–6 mice per group

**Fig. 3:**
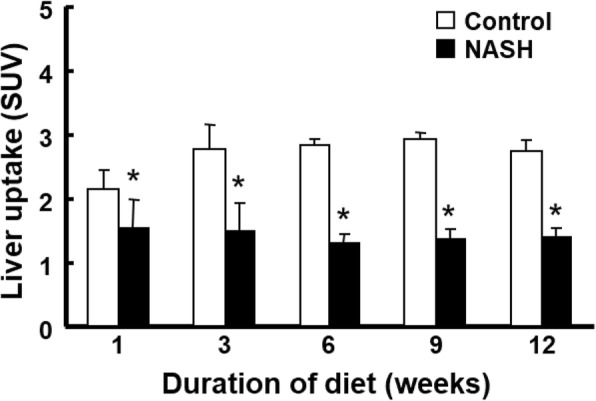
Liver uptake of ^18^F-BCPP-BF in mice fed choline-deficient, l-amino acid defined, high-fat diet and in age-matched control mice. Values represent the summation of standard uptake value (SUV) from 20 to 40 min after injection of ^18^F-BCPP-BF. Data are presented as the mean ± standard deviation of 4–6 mice per group except for the 18-week-old nonalcoholic steatohepatitis (NASH) group, where *N* = 3. *P < 0.05

**Fig. 4:**
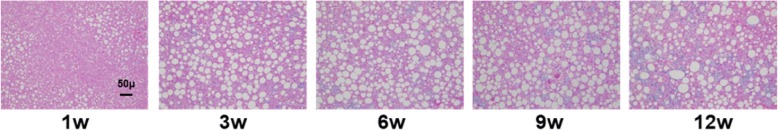
Representative liver histopathology in mice fed a choline-deficient, l-amino acid defined, high-fat diet or a standard diet for 6 to 12 weeks. Images show Masson’s trichrome staining of the liver. Fibrosis was first observed at 6 weeks and became worsened by 12 weeks. Scale bar, 50 μm

**Fig. 5:**
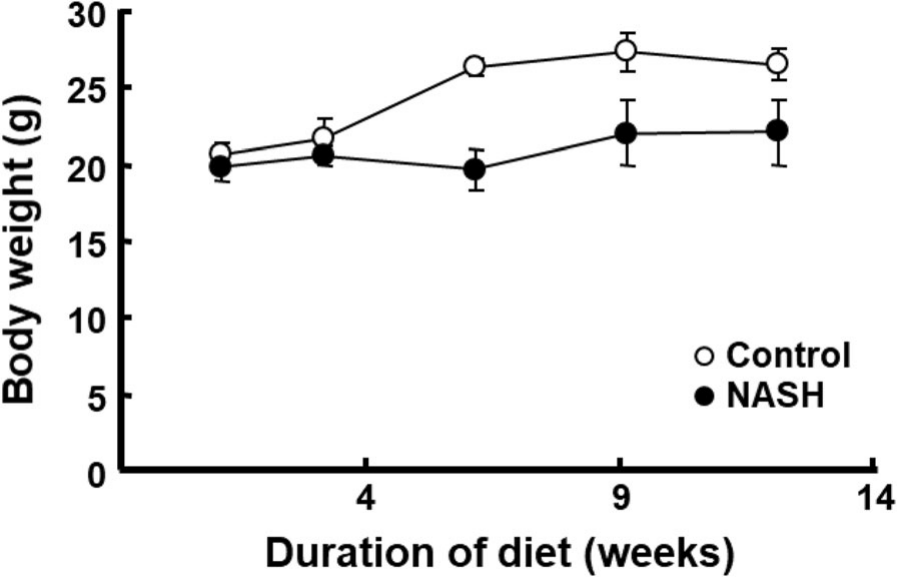
Body weight of mice fed a choline-deficient, l-amino acid defined, high-fat diet (CDAHFD) or a standard diet for 1 to 12 weeks. Data are presented as the mean ± standard deviation of 4–6 mice per group except for mice fed CDAHFD for 12 weeks, where *N* = 3

Summation PET images from 20 to 40 min after injection of ^18^F-BCPP-BF in mice maintained on a normal diet or HFD for 1 to 12 weeks are shown in Fig. 1. Liver uptake of ^18^F-BCPP-BF was significantly reduced after just 1 week and continued to decline up to 6 weeks, at which point it remained at a very low level. The time course of radioactivity concentration (determined from the TAC) in the liver following injection of ^18^F-BCPP-BF into control or NASH mice is shown in Fig. 2. In control mice, concentration of radioactive substance in the liver reached peak values between 20 and 40 min post injection and then slowly decreased. Liver uptake of mice fed normal diet for 1 week was considerably lower as compared to that in aged control mice. In contrast to the pattern in control group, TACs in the liver of NASH mice were almost independent of the duration of exposure to CDAHFD. We carried out a quantitative analysis of the summation of SUV values at the early phase (20–40 min) after injecting ^18^F-BCPP-BF into mice fed CDAHFD for 1 to 12 weeks and age-matched controls (Fig. 3). After 1 week on the diet, liver uptake of ^18^F-BCPP-BF was reduced to 70% of the control value. At 3 and 6 weeks, the reduction was more than twofold lower than in control and remained very low at 12 weeks. NAFLD activity score was obtained from H&E staining (Table 1). After 1 week on the diet, there was accumulation of fat and inflammation in the liver without ballooning. However, by 6 weeks, steatosis, inflammation, and ballooning had progressed and were quite severe. Fibrosis was detected by Masson’s trichrome staining starting from week 6 and rapidly progressed up to 12 weeks (Fig. 4). The body weight of NASH mice on a CDAHFD was lower than that of control mice at each time point, but the degree of weight loss was not as severe as that of MCD diet mice (Fig. 5).

**Table 1:** NAFLD activity score

Duration of diet (weeks)	NASH group					control	
	No. of mice	Steatosis	Lobular inflammation	Hepatocyte ballooning	NAS	No. of mice	NAS
1	4	2.55	1.25	0	3.50	4	0
3	5	3.00	1.80	0	4.80	4	0
6	6	3.00	1.33	1.00	5.33	4	0
9	6	3.00	1.50	1.67	6.17	4	0
12	3	3.00	1.33	2.00	6.33	4	0

*NAS* nonalcholic fatty liver disease (NAFLD) activity score

## Discussion

In this study, a significant reduction of the liver uptake of ^18^F-BCPP-BF in mice fed CDAHFD was observed as early as 1 week after the start of the diet, and the degree of reduction increased with NASH progression. As previously reported, pre-treatment with rotenone (0.1 mg/kg/h), a specific MC-I inhibitor, markedly reduced liver uptake of ^18^F-BCPP-BF in rats [20]. We also reported that 0.1 mg/kg of rotenone fully inhibited the binding of MC-I PET probes to MC-I in the heart and brain [14]. Escalation of rotenone dose to the levels above 0.1 mg/kg/h was almost impossible because of its lethal effect on the cardiac function. In addition, the kinetics of reversible-type PET probes such as ^18^F-BCPP-BF is generally expected to be less sensitive to blood flow changes [26]. In fact, we found the low cerebral blood flow dependence of ^18^F-BCPP-EF, an analogue of ^18^F-BCPP-BF, with reversible-type kinetics in the living brain of stroke-affected [15] and aged [18] monkeys. These results indicated that the reduction of liver uptake of ^18^F-BCPP-BF might reflect decreased MC-I activity rather than diminished hepatic blood flow. Liver uptake expressed as SUV value decreased to about 70% of the control value after just 1 week on the diet (Fig. 3). Although liver uptake in mice fed normal diet for 1 week was considerably lower as compared with that of aged control mice. This might be probably due to increase in hepatic function including MC-I activity during from 6 to 7 weeks of developing process. On the other hand, apparent liver uptake of ^18^F-BCPP-BF in CDAHFD-fed mice seemed to be unaltered with dietary period, which might be due to suppression of MC-I activity. At this stage, mild steatosis and inflammation without ballooning were observed upon histopathological examination. A similar finding of early changes in hepatic mitochondrial function in mice on an MCD was recently reported using ^18^F-BMS-747158-02, another PET probe for MC-I, although in that study, the diet period was only 2 weeks [27]. In that study, both liver uptake of ^18^F-BMS-747158-02 and MC-I activity were measured in liver homogenate, and a high correlation was found between hepatic ^18^F-BMS-747158-02 uptake and MC-I activity. Those observations strongly support our hypothesis that the early and phase-dependent decline in liver uptake of ^18^F-BCPP-BF was due to the impairment of MC-I activity in the liver. The ratio of liver uptake of ^18^F-BCPP-BF in CDAHFD-fed mice to that in age-matched controls decreased to below 50% during the first 6 weeks of feeding and remained at a very low level at 12 weeks. A pathological examination showed that macrovesicular steatosis, inflammation, hepatocellular ballooning, and mild fibrosis were present in mice fed a CDAHFD for 6 weeks, with significant fibrosis persisting longer than the diet period (Fig. 3). The binding specificity of ^18^F-BCPP-BF to MC-1 in the liver was confirmed by pre-administration of rotenone, a selective MC-I inhibitor [20]. Thus, this study provided the first direct measurement of the time course of mitochondrial dysfunction during the progression from liver steatosis to NASH by PET imaging.

A significant reduction in MC-1 activity was observed after just 1 week on CDAHFD. There have been few studies of hepatic mitochondrial dysfunction and ATP homeostasis in the liver during NASH progression [9]. Respiratory activity of MC-I isolated from NASH rats after 3 weeks on an MCD diet was unaltered relative to that in sham animals, but then declined at 7 and 11 weeks. The enhancement of hepatic mitochondrial function during fat accumulation described in that report was not consistent with our current observations. Several factors, such as a different diet model (MCD vs. CDAHFD), species (rat vs. mouse), and differences between in vitro and in vivo settings, could explain the above discrepancy. However, the most likely reason is that PET imaging with ^18^F-BCPP-BF can detect mitochondrial dysfunction in the liver with high sensitivity through direct and high-affinity binding to MC-I. In fact, hepatic mitochondrial dysfunction could be detected using ^18^F-BCPP-BF just 2 h after administration of a low dose of acetaminophen (100 mg/kg) [20].

Fat accumulation caused severe impairment of the mitochondrial function in the liver of rats fed choline-deficient diet [28]. However, NAFLD usually manifests as benign liver steatosis and progresses to NASH in about 25% of cases [29]. Therefore, it is important in future studies to clarify whether benign liver steatosis is also associated with a reduction in MC-I activity in order to validate the effectiveness of ^18^F-BCPP-BF for the diagnosis of NASH at an early phase. It has been recently reported that in vivo mitochondrial redox metabolism in the liver of MCD-fed mice was dramatically altered at an early stage after just 1 week on the diet and that NASH could be accurately diagnosed by in vivo dynamic nuclear polarisation–magnetic resonance imaging using carbonyl-PROXYL as a probe to detect redox changes [30]. In contrast, mice on an HFD did not exhibit significant changes in redox metabolism in the liver; although they showed lipid accumulation at 8 weeks, fibrosis and markers of oxidative stress were not increased relative to their levels in mice on an MCD. These results are similar to our observations: a significant reduction of MC-I activity at an early stage (after 1 week on the diet) detected by PET could reflect the progression of liver steatosis to NASH, although this requires further confirmation using mice fed HFD.

**Fig. 6:**
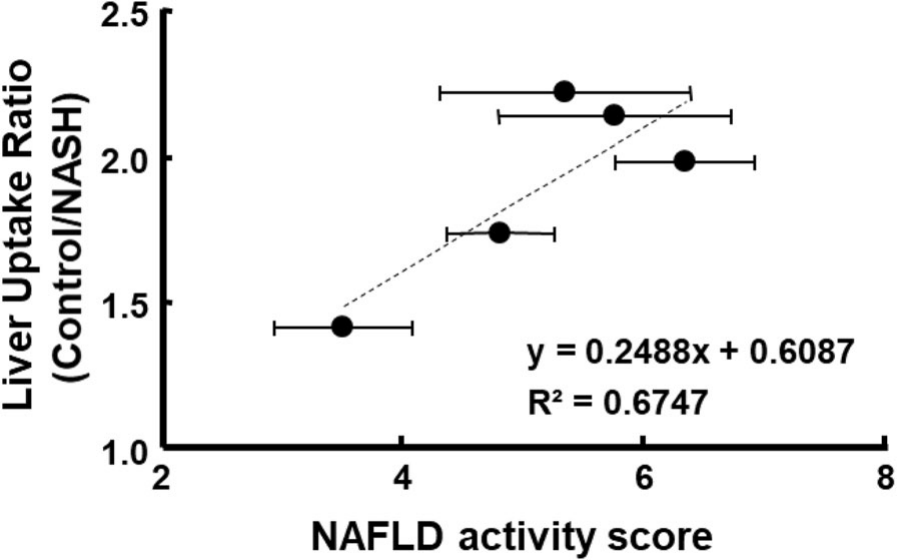
The relationship between the liver uptake ratio (control/nonalcoholic steatohepatitis (NASH)) and nonalcoholic fatty liver disease (NAFLD) activity score of NASH mice

Mitochondrial impairment affects hepatic lipid homeostasis, promotes ROS generation and lipid peroxidation, and induces cytokine release and cell death [8]. The degree of mitochondrial dysfunction estimated based on the reduction of ^18^F-BCPP-BF SUV was more pronounced during 6 weeks of a CDAHFD diet. Along with the histopathological findings during that period, those results indicate that severe mitochondrial dysfunction plays a critical role in the progression of NASH. From 6 to 12 weeks of the diet period, the severity of fibrosis increased, whereas liver uptake of ^18^F-BCPP-BF remained low. The degree of decline in liver uptake was positively correlated with NAFLD scores of CDAHFD-fed mice (Fig. 6), implying that PET imaging using ^18^F-BCPP-BF is useful not only for early diagnosis of NASH but also for disease staging and follow-up of progression.

Although the MCD model is commonly used in basic research, a serious problem with these mice is their dramatic weight loss. The animal model used in the present study is an improved model that rapidly develops fibrosis [23] without excessive weight loss (Fig. 5), enabling a quantitative estimation of ^18^F-BCPP-BF uptake by the liver. For the quantitative kinetic analysis of MC-I binding, the measurements of the correct input function as well as of specific binding in the liver are required; however, these are apparently impossible for several reasons. Full saturation experiment using large amount of MC-I inhibitors cannot be carried out in live animals because of their high pharmacological or toxicological effects. In addition, it is possible that pathological alterations in the liver of NASH model mice changed hepatic blood flow, which affected the kinetics of the PET probe used. Therefore, a semi-quantitative analysis employing SUV values seems to be more practical in both basic research and clinical investigations. Development of the method to estimate the specific binding of ^18^F-BCPP-BF in the liver and other organs is an important subject to be studied in future, and searching of suitable competitive inhibitor is currently under progress.

Reversibility of NASH, including fibrosis, is a major focus of research [31], and efforts to develop therapeutics such as anti-oxidants [11] and molecularly targeted compounds [12] are underway. Monitoring of the efficacy of these compounds in either animal or clinical studies is important for optimising their efficacy. PET imaging is a non-invasive and routinely used method for measuring biochemical processes or functional abnormalities in live animals. Thus, PET imaging with ^18^F-BCPP-BF is valuable not only for the clinical diagnosis of NAFLD/NASH, but also for monitoring the efficacy of new therapeutic compounds in preclinical studies.

## Conclusions

We investigated hepatic mitochondrial dysfunction in mice on CDAHFD from early (1 week on the diet) to severe (12 weeks) stages by PET imaging using ^18^F-BCPP-BF as probe. Importantly, we detected a significant reduction in MC-I activity after only 1 week on the diet, before significant histopathological changes other than steatosis were observed. Mitochondrial dysfunction had worsened by 6 weeks, and a low level of liver uptake of ^18^F-BCPP-BF was maintained for up to 12 weeks. These results provide strong evidence that mitochondrial dysfunction plays a critical role in the progression of NASH. PET imaging using ^18^F-BCPP-BF has high sensitivity for detecting early changes in mitochondrial function in NAFLD and NASH in clinical or research settings. The direct measurement of MC-I activity in the liver by this method can also be useful for monitoring the therapeutic efficacy of novel drugs.

## Abbreviations

^18^F-BCPP-BF: 2-Tert-butyl-4-chloro-5-[6-(4-[^18^F]fluorobutoxy)-pyridin-3-ylmethoxy]-2H-pyridazin-3-one
CDAHFD: Choline-deficient, l-amino acid defined, high-fat diet
H&E: Haematoxylin and eosin
HFD: High-fat diet
MCD: Methionine-choline-deficient diet
MC-I: Mitochondrial complex-I
NAFLD: Nonalcoholic fatty liver disease
NASH: Nonalcoholic steatohepatitis
PET: Positron emission tomography
ROS: Reactive oxygen species
SUV: Standard uptake value
TAC: Time-activity curve
VOI: Volume of interest
